# Medical Items and News of Interest to the Physician

**Published:** 1876-07

**Authors:** 


### For the Use of Physicians.

CULLED FROM THE STANDARD MEDICAL JOURNALS OF THE WORLD.

NOTE.—These formulæ are intended only for the regular practitioner of medicine, and should be employed only by him, or under his immediate supervision.

**Hiccough.**

Dr. Simon reports in the Chicago *Med. Jour. and Exam.*, that a patient who had hiccoughed for twenty-six hours and been subjected to various treatments without success, was relieved almost instantaneously by inhaling three drops of amyl nitric.

**Herpes zoster.**

℞.

Collodion, ℥ i.

Morphiæ muriat, gr. viij. M.

To be painted over the vesicles without breaking them open.

**Goddard's Cosmetic Lotion.**

℞

Tincture Benzoini, f. ʒ ij

Hydrarg-chloridi corrosivi, gr. vi

Aquæ rosæ, f. ℥ vi.

Fiat Mistura.

A valuable cosmetic lotion, but must be used with caution, as it irritates some skins.

**Grapes in Fever.**

Dr. Hartsen, of Cannes (*Centralblatt fur die Med. Wissenschaften*), recommends grapes as a valuable diet in fever.

The grapes contain a considerable amount of hydro-carbonaceous matter, together with a certain quantity of potassium salts, a combination which does not irritate, but, on the contrary, soothes the stomach, and consequently is used with advantage, even in dyspepsia. While considering the carbo-hydrates contained in the grape, we must not neglect the organic acids, particularly tartaric acid. Dr. H. thinks the nourishing influence of these acids too much neglected. It is indeed known that they are changed to carbonic acid in the blood, and are excreted as carbonates in the urine. Possibly careful research might show that, under some circumstances, the organic acids are changed to fats. Dr. H. believes that the organic acids should be ranked with the carbo-hydrates as foods. When fresh grapes are not to be had, raisins or diluted wine might be used.—*Medical Times.*

**A Hard Case.**

Dr. Van Bibber, of Baltimore, reports the case of a gentleman who was in the habit of taking, for eight or ten days consecutively, not less than sixty grains of morphia subcutaneously, together with three pints of whisky and eight or ten cigars. —*Clinic.*

**Anti-Rheumatic Mixture.**

℞.

Potassii nitratis, ʒ j

Vini colchici radicis, f. ʒ j

Spiritus ætheris nitrosi, f. ℥ j

Syrupi guaiaci, f. ℥ ij

Olei gaultheriæ, gtt. vj

Aquæ, q. s. ad. f. ℥ vj. M.

Signa.—Dose, a tablespoonful every two hours. —*Philadelphia Hospital.*

**Offensiveness of Breath and Expectoration.**

Dr. Da Costa, *Medical and Surgical Reporter*, prescribes salicylic acid, five grains, dissolved by means of a drachm of glycerine in half an ounce of water, taken three times a day, in cases where the breath or expectoration are offensive. If internal administration does not accomplish the desired result, it can be used with the atomizer in a solution of similar strength.

**Delirium Tremens.**

Lupuline, in large doses, has proved efficacious in combating the insomnia of delirium tremens. One man, an athlete, had been unable to sleep for ten days, and it was decided to administer to him large doses of lupuline. Three drachms were given in eight ounces of ale every two hours. During eight hours he had taken nine drachms of the drug, and at the end of that time he went to sleep. The usual dose is two drachms every two hours. The remedy has been had recourse to successfully in six cases.—*New York Medical Journal.*

**Cold in the Head.**

Dr. Ferrier, being dissatisfied with the usual formula or treating a cold in the head—namely, "sudorifics and lying in bed"—has made a trial of various substances, to be used in the form of snuffs at the beginning of an attack of nasal catarrh. He finds the following prescription very efficacious:

℞

Muriate of morphia, gr. ij.

Acacia powder, ʒ ij.

Subnitrate of bismuth, ʒ vj.

Of the powder, one-quarter to one-half may be taken as snuff in the twenty-four hours.—DAVID FERRIER, M. D., in *Lancet.*

**Enemata of Chloral.**

M. Dujardin-Beaumetz has employed the process suggested by Griffith for administering chloral enemata, with success. A solution containing a drachm of chloral is beaten up with the yolk of an egg, and this is added to a glass of milk to form the enema. This mixture has the advantage of causing no pain, which is not the case when doses of thirty to sixty grains of chloral are administered in the usual enema or suppository.—*Bull. Gen. de Therap.*

**Hydrophobia.**

Chinese physicians treat hydrophobia in an original manner. Two sandstone bottles half filled with wine or spirits are placed upon a fire until the liquid boils. The contents are then emptied, and the red hot mouth of the bottle is applied to the bite and held there until it is filled with blood, when the same course is pursued with the other bottle. A decoction of rice, in which cantharides have been boiled for an hour and then removed, is also given the patient, who is required to keep perfectly quiet for eight or ten days.

**Pityriasis.**

Dr. Martineau asserts that a solution of forty grains of chloral to an ounce of water, applied to the scalp each morning by means of a sponge, using slight friction, and allowing it to dry, is very efficacious in pityriasis. If the disease is recent, and the lotion is uninterruptedly used for a month, he predicts a certain cure. In the chronic and more obstinate cases, he recommends the continuance of the application of the solution until the disease disappears, as its daily use produces no inconvenience, whilst it releaves the itching.—*Med. and Surg. Reporter.*

**The Eucalyptus.**

A Roman priest named Gildas communicates to the Societe d'Acclimation of Paris, the results of his experiments with the Eucalyptus Globulus near the Monastery of St. Paul Trois Fontaines, in the Roman Campagna. The trees have thriven, and he believes have given indications of their power to purify the atmosphere, though as yet they are not sufficiently numerous to produce much effect. He also reports that a decoction made from the leaves of the tree possesses valuable properties in cases of fever, and that many persons have been cured of that disease by drinking the "elixir," which is also a preventive of fever. A similar preparation of the leaves in the form of a powder is also made, which has the advantage of keeping good longer than the decoction. The opportunity was, no doubt, too tempting for the good monks of St. Paul Trois Fontaines to resist, and we may soon expect to find their "elixir" on the market, and advertised in the religious journals.

*Eucalyptus globulus* has also had its share of attention in India, and without considering the question of the truth or otherwise of its reputed value, it is proved that although it grows quickly and with vigor on the Neilgherries and Khasia hills, at 5,000 to 8,000 feet above the sea, it cannot be induced to live even for a year or two in the hot plains of India.—*The Chemist and Druggist.*

**Ague.**

Dr. Paul Boyce says, in the Virginia *Medical Monthly*:—A case of ague and fever, which had been treated with quinia, arsenic, etc., was cured in four days by simply taking the essence of eucalyptus (prepared from the oil distilled from the leaves) ʒ ij three times a day. I believe we possess in the eucalyptus a remedy not inferior to the cinchona alkaloids. The oil applied to the nerve of a tooth soon destroys its sensitiveness and quiets the pain. In purulent catarrhal affections of the urethra it acts like a charm.—*Med. and Surg. Reporter.*

**Neuralgia.**

℞.
Acidi arseniosi, gr. iv
Strychniæ sulphatis, gr. iij
Extracti belladonnæ, gr. xxiv
Cinchoniæ sulphatis, ʒ iiij
Pilulæ ferri carbonatis, ʒ v. M.
Fiat pilulæ cxx.

Signa.—Each pill contains 1-30th grain of arsenic, 1-40th grain of strychnia, 1-5th grain of belladonna, 1½ grains of cinchonia, and 2½ grains of Vallets mass.—*From the Pharmacopœia of Philadelphia Hospital.*

**Detecting Sugar in Urine.**

In the new edition of his work on Diabetes, Dr. J. Seegen recommends a modification of Trommer's test, which consists in filtering the suspected urine two or three times through animal charcoal, and then washing out the latter with a very little distilled water. To this wash-water Trommer's test is applied in the usual way. Very small quantities of sugar can be detected with certainty thus, for the wash-water is generally as sensitive to the test as an aqueous saccharine solution—that is, much more sensitive than the corresponding urine, in which the reaction of the sugar is impaired by the presence of other constituents.—*Med. Times and Gaz.*

**Skin Diseases.**

Dr. Maccormac recommends petroleum for porrigo and favus in preference to other remedies. The plan of treatment, so far as the scalp is concerned, is to clip or shave the hair closely. One part of petroleum is to be added to two of lard. This ointment should be applied gently, but thoroughly, once or twice a day. In cases that have been neglected, poultices of bread or linseed meal should be used before applying the ointment. After the application, a piece of dry, soft linen rag may be laid on, and over all a clean linen cap. Before the next application of the ointment, the head must be cleansed with black or fish soap, and warm water.—*The Practitioner.*

---

**Rheumatism.**

A correspondent of the *Scientific American* writes to say, as a counterblast to an article on the wonderful efficacy of ammonia in this painful affection, that caustic ammonia is no infallible cure for rheumatism. "I read your paragraph on this subject on the day on which I was treated to the novel experience of acute rheumatism in my left leg. Several drops of ammonia were taken at once without effect, and similar doses a few days later. The complaint has steadily grown more aggravating since.

"This rheumatic experience of mine also demolishes a popular fallacy, to which I admit I gave credence, that perfectly abstemious habits act as a prevention of such afflictions."

---

**Mead.**

The following formula, given by Prof. E. S. Wayne, at the Pharmaceutical Meeting of the Cincinnati College of Pharmacy, is kindly communicated by Mr. Louis Schwab, the corresponding secretary. It will prove, no doubt, timely and useful to our readers:

| | | |
|---|---|---|
| Sarsaparilla root, contused, | | |
| Licorice root, | " | of each, 8 ozs. |
| Cassia bark, | " | 8 " |
| Cloves, | " | 2 " |
| Coriander seed, | " | 3 " |
| Ginger, | " | 8 " |

Boil for fifteen minutes in eight gallons of water; let it stand until cold, or for several hours, to settle down. Then strain through flannel, and add to it, in the fountain:

Syrup, 12 pints;

Honey, 4 ounces;

Tincture of ginger, 4 ounces;

Solution of citric acid, 4 ounces;

Add enough water to complete ten gallons, and charge with gas.

**Tea Poultice.**

Dr. B. G. McVail, in the *Virginia Medical Monthly*, writes from Camp Douglas, Utah, a military post of the United States army, that an accidental circumstance led to apply a *tea poultice* to a case of erysipelas of the hand and forearm, and with such happy results that he has since used it in several cases—also for whitlow and osteitis. In every case it gave decided satisfaction. It is grateful to the patient, allays pain, and reduces inflammation.

Flaxseed meal made into a poultice with a strong decoction of *black tea* is a nice way to apply it, or the decoction may be applied warm on cloths. His opportunities for using it have been somewhat limited, he says, and does not justify him in speaking too positively of its merits; but having been so much pleased with its action, he is induced to request a trial by the profession in the above affections and in puerperal peritonitis. Tannin has not given the same results in its pure state.

---

**Specific against Hydrophobia.**

Dr. Grzyvala of Krivoe Ozero, Podolia, for whose trustworthiness Professor Gubler of Paris vouches, declares that, after a series of crucial trials which he describes at length, he has found that, after having had opportunities of treating at least one hundred cases of men bitten by rabid dogs, with the *Xanthium Spinosum*, he has never in any one of these cases failed to ward off hydrophobia. He gives some startling examples. During the Crimean war, a family of twelve persons had been bitten by a hydrophobic wolf. Six of them entered his wards in the hospital of Olschanka, government of Podolia, district of Balta. They were treated with infusion of the leaves of Xanthium, and all recovered. The six others, who were treated by the actual cautery and the daily use of genesta tinctoria and other drugs, died with hydrophobia in the course of twelve to sixty days. He recounts many other facts not less striking. For an adult, the dose is sixty centigrammes of the dry powder, repeated three times a day, and continued during six weeks. Children under twelve take half that quantity. The dose for animals is much larger. A herd of thirty oxen had been bitten by a mad wolf; eight had succumbed with symptoms of hydrophobia. The Commissary of Police came to Dr. Grzyvala for his "antirabic powder." He gave three ounces of the powder, with bran, daily to each of the remaining animals; none of them suffered from the disease. These are examples of which Dr. Grzyvala says he has a hundred others.—*Br. Medical Jour.*

**Typhoid Fever.**

Dr. T. J. Printer, in a letter to the November number of *St. Louis Med. and Surg. Journal*, from Leipsic, speaks of the use of salicylic acid for the treatment of typhoid fever, in the wards of the hospital, which are under the direction of Prof. Wunderlich. The results are described as remarkable. The most striking and uniform effect was a fall in the temperature within twenty-four hours after the administration of the remedy. The dose was one gramme (fifteen grains), in solution in water, repeated three or four times during the day. The average decrease in the temperature is stated to be about three degrees Fahr., though it sometimes fell as much as from 104° to 99° Fahr. The fall of temperature was accompanied by a like amelioration of all the other symptoms. Prof. Wunderlich, in speaking of the value of the remedy, declined to express himself definitely as to its merits, "but mentioned the fact of the fall of temperature as full of promise for its usefulness." It had only been on trial for two months.

---

**Diabetes Mellitus.**

Dr. Julius Jacobs ( *Virchow's Archiv.* v. 65, p. 481 ) alludes to the futility of all previously lauded means of treatment in this affection, and gives notes of two cases treated by Shultz's method. Shultz maintained that diabetics lose, by excessive excretion of sugar, enormous quantities of respiratory material, and are obliged to give up their fat and protein for his purpose. If glycerine, which is not changed into sugar in the organism, but is transformed directly into carbonic acid and water, be administered, this waste is compensated, or rather vicariously prevented, by the glycerine. Jacobs made use of the following formula:

℞

Glycerinæ, ℨ vi.

Pulv. acid. tartaric, ℈ iv.

Aq. destillat, f ℥ xss.—M.

To be taken in twenty-four hours.

Although Jacobs did not obtain very brilliant results from this treatment, yet the patient's improvement was sufficient to encourage further use of the remedy. The usual food was taken.

---

**Medicated Ice.**

The possibility of using frozen antiseptics in medicine was recently pointed out by Mr. Edward Martin, in a letter to the *Lancet*, as follows: "Every practitioner has at times to face the difficulties of the scarlatinal throat in young children. It may sadly want topical medication; but how is he to apply it? Young children cannot gargle, and to attempt the brush or the spray often fills them with terror. In many cases neither sternness nor coaxing avails. Yet these little ones in almost every case will greedily suck bits of ice. This has long been my chief resource where I could not pursuade the child to submit to the sulphurous-acid spray. Lately I have been trying an ice formed of a frozen solution of the acid (or some other antiseptic). Though of course not so tasteless as pure ice, the flavor is so much lessened by the low temperature, and probably also through the parched tongue very little appreciating any flavor whatever, that I find scarcely any complaint on that source from the little sufferers; they generally take to it very readily. The process of making it is very simple; a large test-tube immersed in a mixture of pounded ice and salt is the only apparatus required, and a momentary dip of the tube in hot water enables one to turn out the cylinder of ice as the cook turns out her mould of jelly. I have tried the three following formulæ, all of which answer, though I think I prefer the first:

1. Sulphurous acid, half a drachm; water, seven drachms and a half: mix and freeze.

2. Chlorate of potassium, one scruple; water, one ounce: dissolve and freeze.

3. Solution of chlorinated soda, half a drachm; water, one ounce: mix and freeze.

However, the form is of secondary importance, as each practitioner can construct his own. Boracic acid, salicylic acid, or any other harmless antiseptic with not too much taste, would, doubtless, be as useful as those indicated.

---

**Bowel Affections.**

Dr. M. J. Eley, of La Fayette, Ala., writing to *The Medical and Surgical Reporter*, recommends the following preparation as being a safe and efficient remedy. He says: "In the number of your journal for September 7th, 1872, Dr. Downing called attention to the following "New combination for bowel affections," asking other physicians to try it and report the result.

The formula is as follows:

℞.

Socotrine aloes, powd.,

Sulph. potash, powd.,

Bicarb. soda, āā. ℨ j

Cloves, powd., ℨ ss. M.

Divide into twelve powders.

To one of these powders he added three tablespoonfuls of boiling water, giving the whole at one dose—as hot as could be borne—to an adult. To be repeated every hour. To children one teaspoonful to be given every half hour.

The remedy was warmly recommended in all cases of cholera morbus, diarrhœa, and dysentery.

Since I first noticed the above mentioned combination, now four years, I have been using it, with uniform satisfaction, in the above named troubles as seen in children, especially in what we usually term "summer complaints" and "teething diarrhœa." My plan of administering is somewhat different from Dr. Downing's; for instance, I add one teaspoonful of the powder to a teacup of boiling water; when cool, give a teaspoonful every hour till the discharges become normal in frequency and color.

**Otorrhœa.**

Dr. Thomas G. Morton, of Philadelphia, writes to the *Medical Times* that he has used styptic cotton with excellent results in a number of cases of discharge from the ears.

The styptic cotton is prepared as follows: Take a roll of fine jeweler's cotton and thoroughly saturate it in a mixture of Monsel's solution of the subsulphate of iron, diluted with two parts of water; let it stand in the mixture for forty-eight hours; press the liquid out, and dry in a warm room, then pick or card out in fine shreds. It is better to make it in small quantities, as there seems to be some change in the cotton when kept for any length of time; losing its texture and breaking up in a fine powder when handled, thus rendering it unfit for application. In the case cited, the first plug was removed the day following its insertion showing a healthier condition of the meatus, with marked lessening of the discharge; all the pus was absorbed by the cotton, which left the drum and canal quite clean; at the end of the first week only a slight moisture remained, and in a fortnight the cure was complete, and has so continued; the opening in the tympanum closed up at the same time.

Encouraged by success in this instance, he has since used no other treatment in a very large number of cases of otorrhœa, and always with the same excellent result.

Before using the cotton, the ear should each time be thoroughly cleansed with tepid water, with Davidson's syringe, and the canal then wiped out with common cotton; then a portion of the "styptic cotton" should be rolled upon an instrument having a screw shaped extremity, an illustration of which accompanies the original article. The plug of cotton being at least the length and width of the canal and carried in until the plug rests close to, but not in contact with, the drum; by reversing the screw movement the instrument is easily withdrawn, leaving the cotton behind.

The first few plugs should be changed daily, then, according to the lessening of the discharge, not so frequently.

He has not observed any trouble or pain arise from the use of the styptic cotton, while it usually has an anodyne effect.

[In preparing this cotton, a precaution not mentioned by Dr. Morton consists in carefully cleansing it with caustic soda to remove adherent oil, and subsequent washing to get rid of the alkali.]

**Fancy Soda Water Syrups.**

*Catawba.*—Prepare a very heavy simple syrup, using sixteen pounds of sugar to a gallon of water. Add to a portion of this an equal bulk of fine catawba wine having a rich bouquet. Hock, claret and other wine syrups are prepared in a similar manner. Some dealers prefer to keep the simple syrup on draught, and after drawing a sufficient quantity in the glass to properly sweeten the contents, add the wine just previous to filling with soda water.

*Milk Punch.*—To one pint of heavy syrup add a half pint each of brandy and Jamaica rum. Flavor with two teaspoonfuls of an extract prepared by macerating two ounces of ground nutmegs in eight ounces of alcohol. The syrup is first to be poured into the glass in the proper quantity, and ordinary cream syrup added before drawing the soda water.

*Champagne.*—To one quart of good Rhine wine, with a rich bouquet, add two ounces of old Otard brandy, one tablespoonful of good sherry, and three pounds of pulverized sugar. Dissolve the latter by stirring without the application of heat. This syrup should be kept very cold, and used with soda water from a highly charged fount. It forms a much better substitute for genuine champagne than any of the numerous doctored cider imitations.

*Sherry Cobbler.*—To one pint of good sherry add an equal measure of heavy simple syrup and one lemon cut in very thin slices. Allow the syrup to stand a few hours, strain through a sieve, and bottle for use.

*Compound Syrups.*—Under the names of Ambrosia, Nectar, etc., many variously flavored combinations are from time to time set forth by the dealer to give variety to his list of syrups, and attract custom by their novelty. As no special uniformity need be observed in this regard, a few formulas are given, to any of which the dealer using may assign that name which his fancy may suggest.

I.—Take of simple syrup one pint, syrup of wild cherry and good port wine, of each four ounces. The flavor of this is exquisite when made from fine materials.

II.—Raspberry syrup one pint, vanilla syrup one pint, Sauterne wine one half pint.

III.—Vanilla syrup one quart, pineapple syrup one half pint, raspberry syrup one half pint.

IV.—Heavy simple syrup one pint, lemon syrup one half pint, brandy one half pint, extract of nutmeg ten drops.

V.—To one quart of good Rhine wine add one lemon and one orange, each thinly sliced, one half of a small pine apple, sliced, and three pounds of fine sugar. Allow them to stand a few hours, heat gently over a water bath, stirring until the sugar is dissolved, and strain for use.

---

**Diabetes Treated by Skimmed Milk.**

Dr. Donkin read the reports of two cases, the subject of one of which, a carpenter, was present for examination. This man had fallen from a cart in June last, and been dragged 400 or 500 yards. Shortly afterwards he began to suffer from the usual symptoms of diabetes. On October 6th he was first seen by Dr. Donkin, who found that the quantity of urine voided in twenty-four hours was about twelve pints, that its specific gravity was 1045, and that it contained twenty-eight grains of sugar to the ounce. There was great general prostration. Next day the patient's diet was restricted to seven pints of skimmed milk daily, being increased to eight pints a day on October 9th. The specific gravity of the urine decreased at once, and on October 14th was 1010, whilst the urine did not contain a trace of sugar. There was a consentaneous great improvement in the general condition of the patient. Shortly a mutton chop, with cabbage, was allowed daily, then twice daily, and subsequently with fish, sugar being still absent from the urine. Lastly, tea and a little brandy were added to the daily diet. Dr. Donkin considered this case another corroborative proof of his position, that milk-sugar, as a constituent of milk, does not contribute to the formation of diabetic sugar. The second case was that of a child, ten years of age, of healthy parentage. She had been very healthy until about July last, when all the usual symptoms of severe diabetes began. She was then placed, by her usual medical attendent, on a meat diet; iron was given as a tonic, and Dover's powder at bedtime. Slight improvement followed this treatment. On October 17th, the skimmed milk diet was begun, and, in a week, the specific gravity of the urine had fallen from about 1040 to 1016. On October 29th, the density of the urine was 1012, whilst it did not contain a trace of sugar. Sir Thomas Watson had been consulted in the case before the treatment was begun, and had received from the patient's father a specimen of urine passed after the treatment had been adopted for eighteen days; and he wrote that it had a specific gravity of 1015, and that it did not contain a trace of sugar, nor any albumen. Her diet was then increased by the addition of beef-tea, mutton-chops, and cabbages. Her general health greatly improved. Dr. Donkin thought these two cases good illustrations of the efficacy of the treatment in arresting the disease when applied early.

Dr. Glover remarked on the interest and importance of the cases related. He suggested that the man, who was present, should have his urine examined for sugar. This was done, and none was found.—*London Medical Times.*

---

**Rapid Cure of Acute Rheumatism with Salicylic Acid.**

The first number of the *Berliner Klinische Wochenschrift* for the new year (we quote from the *Boston Med. and Surg. Journal,*) contains an article on the treatment of polyarthritis rheumatica with salicylic acid, containing such interesting statements, and emanating from so high an authority as Professor Traube, that we hasten to give some account of it to our readers. The cases occurred in Traube's clinic, and are reported by one of his assistants, Dr. Stricker. The latter states that for several months past all the patients affected with acute rheumatism, in whom the local symptoms were well marked, have been treated with salicylic acid, and that in all cases at the end of forty-eight hours, frequently much sooner, there was not only a return to the normal temperature, but also a complete disappearance of swelling, redness, and particularly of pain in the joints. Dr. Stricker proclaims boldly that the experience which he has obtained in these cases cannot be attributed in any way to chance, and the salicylic acid must, therefore, entirely apart from its antipyretic action, be looked upon as having a specific action on acute rheumatism and affording a means of the radical cure of the disease.

It is quite important that a pure pulverized acid should be obtained. When carefully prepared and pure, the crystals are very white and glistening needles, without smell, and soluble without cloudiness in water and alcohol. In this form it can be given in large doses without disturbing the digestion. It is administered in a wafer, to protect the mucous membrane of the mouth and pharynx from dryness and burning, which sometimes follow contact with the crystals. The dose is from one-half a gramme to a gramme, or seven and one-half to fifteen grains, and is given every hour until the diseased joints can be moved without pain. To effect this it may be necessary to administer as much as fifteen grammes, never less than five grammes. This treatment produces increased perspiration, tinnitus aurium, and deafness, and rarely some mental exhilaration, symptoms which hardly contraindicate its use. The treatment should be begun early in the morning, and is occasionally complete before night. In one case an enthusiastic patient took, without leave, twenty-two grammes in the space of twelve hours without the slightest gastric disturbance. Meanwhile the tongue, which was heavily coated, had cleaned up, and the lost appetite returned. The effects of the drug upon the secondary inflammations, particularly those of the pericardium, have not yet been sufficiently studied, the material for that purpose not having been adequate. The whole number of cases thus treated is but fourteen; the effects of the treatment are so uniform, however, that the profession is strongly urged to try it. This method has, we believe, also been carried out by another Berlin physician with success. The first of Stricker's cases only is given in the number of the journal to which we have referred; the account of the others will follow. In this case there was considerable elevation of temperature, while several phalangeal joints of the left foot, the left knee, the shoulder, and the wrist were slightly reddened, swollen, and exceedingly painful. The patient had been sick several days; treatment was begun in the evening, and by the following afternoon all the joints were well, while his appetite had returned with great vigor.

Although the data are far from sufficient to establish the claim made for the drug in the treatment of the disease in question, the name of Professor Traube furnishes an indorsement to the facts which does not permit them to be treated lightly. We shall await with much interest the report of other cases and the results of a general trial of the remedy, which will be sure to follow this somewhat startling announcement. In the mean time it would be well, keeping in mind the experience with regard to the "Lostorfer corpuscles," not to let our expectations be raised too high.

In the same paper, (the *Boston Med. and Surg. Journal,*) is a report of a case from Dr. Charles P. Putnam, which is interesting as supporting the rapid and excellent effects of salicylic acid in acute rheumatism. The case is that of a child five years of age; the history is as follows:

Alice B., five years old, was thoroughly chilled while skating on February 5th, and for several days after had a hot skin, a coated tongue, and no appetite. On the 9th she lay on the bed most of the day. On the 10th she remained in bed. On the 11th, in the morning, her pulse was 120, her temperature (axillary) 102.7°. In the evening, pulse 131, temperature 103.5°. During the latter part of this day she complained much of pain in the muscles of the thigh, in the groin, and in the ankles. She would not move her lower extremities, and could be moved only with great pain. Skin dry. No swelling nor redness of any joints or other parts of the body. On the 12th the general state was as before; pulse 120, temperature 103°. During the day the hands became painful and sensitive, and the knuckle-joint of the third right-hand finger was slightly swollen and rose colored.

On the 13th, pulse 130, temperature 102.7°. She had slept somewhat, with five grains of Dover's powder, but the pain and tenderness of the extremities had increased very much, so that the patient lay quite helpless, cried out when touched, and was moved only with the greatest difficulty. Most of the joints of the hands and feet, especially the joints between the first and second phalanges of the right hand and foot, were swollen and rose-colored. Movement of the jaws also caused pain.

Salicylic acid was prescribed, five grains in wafers every hour when the patient was awake. The treatment was begun at 2 P. M., and eleven doses making almost one drachm, had been given at 10 A. M., next day. No tinnitus aurium was observed by the patient. After the first three doses there seemed to be some improvement, for though the physical appearance remained unchanged, the patient's spirits were better.

After the seventh dose she went to sleep, and was quiet most of the night, only waking often enough to take two more doses. In the morning she turned over in bed, moved the bedclothes with her hands, and wanted to be dressed.

On examination it was found that the redness had left all the joints; they could be moved without pain, and only a moderate œdematous swelling marked the affected parts. The patient complained only of slight itching of the hands and face.

At 10 A. M., pulse 120, temperature 101.6°. At 6 P. M., pulse 124, temperature 102.4°. Five grains of salicylic acid were ordered every two hours during the night, but only three doses were given, as the patient seemed quite comfortable and slept. On the 15th, (forty-eight hours after treatment began), the temperature was 99.9°.

